# Quadruple H therapy for vasospasm

**DOI:** 10.4103/0972-2327.48847

**Published:** 2009

**Authors:** M. Sami Walid, Nadezhda V. Zaytseva

**Affiliations:** Medical Center of Central Georgia, Macon, USA; 1 Kuban State Medical University, Krasnodar, Russian Federation.

**Keywords:** Hyperbaric oxygenation, vasospasm

## Abstract

Triple H therapy has been enthusiastically used to increase cerebral blood flow in cases of vasospasm. Nevertheless, the oxygen-carrying capacity of the blood is lowered with this treatment. This side effect can theoretically be partially corrected using hyperbaric oxygen therapy (HBO) which appears to be the missing ring in the above therapeutic regimen. We conducted a review of of the available evidence regarding the beneficial effects of HBO in preventing postoperative ischemic complications due to vasospasm after surgery on ruptured cerebral aneurysms and the rationale for including HBO into the standards of care of these difficult patients.

Delayed cerebral ischemia due to vasospasm is a major cause of death and disability in patients with subarachnoid hemorrhage (SAH) set off by a ruptured intracranial aneurysm. Different treatment modalities to combat vasospasm were used over the last four decades. Wise *et al*,[1] showed that neurological deficits could be reversed by administering vasopressors with improvement in neurological status noted when systolic blood pressure was raised to 150–170 mm Hg and diastolic pressure raised to 85–100 mm Hg. Several years later, Kosnik and Hunt[2] and Giannotta *et al*,[3] were able to reverse neurological deficits after ischemia due to vasospasm by elevating central venous pressure with whole blood, plasma, or albumin. Further efforts to combat this disorder came when Allen *et al*,[4] showed that calcium antagonist, nimodipine, reduced the occurrence of severe neurologic deficits due to cerebral arterial spasm.

Triple H therapy has been enthusiastically used to increase cerebral blood flow in cases of vasospasm. The ischemic deficits were shown to partially resolve with this regimen. Therefore, it was applied prophylactically against delayed ischemic deficits in all patients with SAH. Nevertheless, aggressive use of this therapy led to the recognition of its deleterious effects. Shimoda *et al*,[5] studied 323 patients with SAH among whom 112 patients developed delayed ischemic deficit, 94 of whom underwent hypervolemic therapy. Infarction due to vasospasm was found ultimately in 43 of these 94 patients. Twenty six patients (28%) developed intracranial complications during hypervolemic therapy – cerebral edema was aggravated in 18 patients, and hemorrhagic infarction developed in 8 patients. A more cautious approach is now advocated. The relative contributions of the components of this therapy to its efficacy are still a subject of debate.

Hypervolemia and hemodilution, although help increase cerebral blood flow due to decreased viscosity, lowers the oxygen-carrying capacity of the blood. This side effect can be partially corrected using hyperbaric oxygen therapy (HBO) which appears to be the missing ring in the above therapeutic chain. The first suggestion that raised air pressures might be used in the treatment of human illness was made in 1664 by Henshaw in England.[6] The first hyperbaric chamber to investigate the therapeutic action of compressed air on human body was described and built by the Swiss physician Junod in 1834.[6] Using 1.5 atmospheres of pressure, Junod was reported to have treated paralytic patients with beneficial results. This pioneering work was not continued until Ingevar and Lassen[7] demonstrated positive results in four patients suffering from focal cerebral ischemia. Since then, numerous articles have been published demonstrating that HBO is useful for the treatment of both acute and chronic stroke. Neubauer and End[8] reported using HBO as an adjunctive therapy in 122 patients with strokes due to thrombosis. HBO has also been shown to be beneficial in preventing repeated stroke. Pravdenkova *et al*,[9] compared the course of the acute period of cerebral stroke in two matched groups of patients – the basic group (*n* = 124) receiving HBO and the control group (*n* = 108) receiving no such treatment. It was found that one (0.81%) patient of the basic group developed recurrent impairment of the cerebral circulation, whereas in the control group this complication occurred in 10 (9.26%) patients. Isakov *et al*,[10] included HBO in the complex of therapeutic measures applied in the postoperative period in patients with ruptured aneurysms of the cerebral vessels. From comparison of the course of the disease in 47 patients treated by HBO with that in 30 patients not subjected to HBO, the authors concluded that the inclusion of HBO in the complex of measures applied after operations on the cerebral vessels for ruptured aneurysms had a positive effect on the course of the disease – the critical condition was shorter; the duration of the meningeal syndrome, headache, and temperature reaction was less by six days than in the control group; the number of patients with good treatment results increased by 18%; mental disorders in the absence of hematoma of the frontal area were prevented; and the frequency of postoperative wound infections were reduced. Similar results have been reported by Kohshi *et al*,[11] who evaluated the efficacy of HBO therapy in 43 patients who developed symptomatic vasospasm following acute aneurysm surgery. They found that adjunctive HBO significantly reduced the incidence of infarcts in patients who developed symptomatic vasospasm after acute aneurysm surgery. Levina *et al*,[12] studied HBO effects on the course of acute cerebral ischemia due to vasospasm after early surgery for ruptured cerebral aneurysms in 190 patients – 110 patients received HBO séances in the complex therapy of the postoperative period at 1–5 days after aneurysm clipping and 80 similar patients were treated without HBO. Positive dynamics of the clinical picture were observed in the majority of patients after HBO séances that were associated with decreases in the size of the brain infarct and perifocal brain edema on CT. As results of transcranial dopplerography demonstrated, HBO treatment did not intensify vasospasm severity and normalized cerebral blood flow in half of cases. According to the results of associated electroencephalography, HBO had positive effect on brain electrical activity. Postoperative complications in the HBO-treated group happened more rarely compared with the control group. There were positive effects on the clinical and physiological parameters when HBO treatment was used as an adjunct to the complex therapy of acute cerebral ischemia in the early treatment of ruptured intracranial aneurysms. The peak of action of the therapeutic effect of HBO in ischemia is speculated to be in the penumbral area, thanks to cerebral blood flow normalization.

A sound physiological and anatomical evidence for the mechanism by which HBO improves acute and chronic stroke and brain-damaged individuals has accumulated over the past four decades. The concept of an ischemic marginal zone surrounding a central core of infarcted brain tissue as a component of stroke-induced damage was introduced by Astrup, Symon, Branston, and Lassen.[13] Their baboon studies showed that electrical activity was lost at the periphery of a cerebral infarct when the blood flow fell below 15 ml/100 g/min while neuronal death began to occur when blood flow fell to 6–8 ml/100 g/min. These low blood flow values may be used to define an area surrounding an infarct where the tissues remain alive but lose their function called the ‘ischemic penumbra.’ The ischemic penumbra is defined as “an area of moderately ischemic brain tissue surrounding an area of more severe ischemia; blood flow to this area may be enhanced in order to prevent the spread of a cerebral infarction.”[14] It is a dynamic process of impaired perfusion and metabolism propagating from the center of ischemia to the neighboring tissue.

Repeat multitracer PET studies on human stroke victims performed by Heiss *et al*,[15] have shown viable tissue in the borderzone of ischemia up to 48 hours after the cerebrovascular attack. With few exceptions, these tissues suffered progressive metabolic derangement and had decreased cerebral metabolic rates of oxygen within two weeks after the stroke, −17.2% vs −26.1%, as compared to normal mirror image regions of interests. For many years cerebral ischemia has been thought to release glutamate from the hypoxic-damaged cells and this glutamate was thought to potentiate and propagate the initial hypoxic damage. An alternative explanation for glutamate-mediated injury was described by Hossmann[16] based on hypoxia and peri-infarct spreading of depression-like depolarizations. These irregular depolarizations are thought to initiate or worsen hypoxic episodes due to energy expenditures and cause further suppression in protein synthesis, gradual deterioration in energy metabolism, and progression of irreversibly damaged tissue into the penumbra zone. He stated that “interventions to improve ischemic resistance should therefore aim at improving the oxygen supply or reducing the metabolic workload in the penumbra region.”

HBO is the only treatment that is able to do this task. It consists of 1-2 séances a day in a sealed chamber called ‘barocamera’ for a total of 6–15 séances. The séance lasts from 45 to 120 minutes during which the person's body is surrounded by air pressure equivalent to the pressure produced by diving 16–33 feet underwater equal to 1.5–2 atmospheres absolute (ATA). During HBO séance, the patient is under constant visual observation and electrocardiographic monitoring. By raising the arterial levels of oxygen 10–15 times higher than that corresponding to normal atmospheric pressure, the elevated oxygen pressure within the organism exerts therapeutic benefits on acute and chronically ischemic tissues. Adding vitamin C, a potent antioxidant, helps reverse the possible endothelial dysfunction from increased oxidant stress.[17] Prices are widely different from one country to another. For example, an HBO séance in the US costs around USD200; in Russia it costs around 300 Rub. ($10).[18,19]

In the setting of cerebral vasospasm, in addition to triple-H therapy, angioplasty may be performed to reverse or limit brain ischemia. This technique, first introduced by Zubkov *et al*,[20] can be used acutely to increase the caliber of the arteries affected by spasm. Endovascular infusion of vasodilatatory substances was first performed by Kaku *et al*,[21] who performed intra-arterial papaverine infusion in 10 patients with vasospasm. Recently, the calcium channel blocker, verapamil, was efficiently used endovascularly in the treatment of vasospasm.[22]

The goal of this paper is to promote awareness about HBO as a promising treatment modality in vasospasm. HBO is one of those medical subspecialties that unfortunately are left behind by the biases of modern medical science which is primarily based on pharmaceutical economics. From the above review we see that the evidence for the beneficial effects of HBO in preventing postoperative ischemic complications due to vasospasm after surgery on ruptured cerebral aneurysms is provocative. HBO appears to be an integral part that is missing in the therapeutic regimen for vasospasm. HBO can empower current pharmaceutical and surgical management with higher levels of treatment success bringing cures where partial successes are the norms. HBO has already demonstrated good clinical success in incurable diseases, including stroke, cerebral palsy, radiation necrosis, and other conditions. While modern medicine debates the etiologies and optimal treatment protocols, curative HBO applications should be used today. The application of the quadruple H therapy – Hypertension, Hypervolemia, Hemodilution, and Hyberbaric oxygenation – appears to have better curative perspectives with minimal additional cost. Multicenter, randomized clinical trials will provide level-1 evidence about the superiority of quadruple H therapy in vasospasm in order to incorporate HBO into the standards of care of these difficult patients.

## References

[1] Wise G, Sutter R, Burkholder J (1972). The treatment of brain ischemia with vasopressor drugs. Stroke.

[2] Kosnik EJ, Hunt WE (1976). Postoperative hypertension in the management of patients with intracranial arterial aneurysms. J Neurosurg.

[3] Giannotta SL, McGillicuddy JE, Kindt GW (1977). Diagnosis and treatment of postoperative cerebral vasospasm. Surg Neurol.

[4] Allen GS, Ahn HS, Preziosi TJ, Battye R, Boone SC, Boone SC (1983). Cerebral arterial spasm: A controlled trial of nimodipine in patients with subarachnoid hemorrhage. N Engl J Med.

[5] Shimoda M, Oda S, Tsugane R, Sato O (1993). Intracranial complications of hypervolemic therapy in patients with delayed ischaemic deficit attributed to vasospasm. J Neurosurg.

[6] Jain KK (2004). Textbook of Hyperbaric Medicine. 4 Rev Exp edition.

[7] Ingvar DH, Lassen NA (1965). Treatment of focal cerebral ischemia with hyperbaric oxygen: Report of 4 cases. Acta Neurol Scand.

[8] Neubauer RA, End E (1980). Hyperbaric oxygenation as an adjunct therapy in strokes due to thrombosis: A review of 122 patients. Stroke.

[9] Pravdenkova SV, Romasenko MV, Shelkovskii VN (1984). Hyperbaric oxygenation and prevention of recurrent cerebral circulatory disorders in the acute stage of a stroke. Zh Nevropatol Psikhiatr Im S S Korsakova.

[10] Isakov IV, Pravdenkova SV, Shchelkovskii VN (1985). Hyperbaric oxygenation in ruptured cerebral aneurysms during the postoperative period. Zh Vopr Neirokhir Im N N Burdenko.

[11] Kohshi K, Yokota A, Konda N, Munaka M, Yasukouchi H (1993). Hyperbaric oxygen therapy adjunctive to mild hypertensive hypervolemia for symptomatic vasospasm. Neurol Med Chir (Tokyo).

[12] Levina OA, Romasenko MV, Krylov VV (2002). Therapeutic effects of hyperbaric oxygenation (HBO) on acute cerebral ischemia in patients after intracranial aneurysms clipping. Eur J Underwater Hyperbaric Med.

[13] Astrup J, Symon L, Branston NM, Lassen NA (1977). Cortical evoked potential and extracellular K+ and H+ at critical levels of brain ischemia. Stroke.

[14] (1994). Dorland's Illustrated Medical Dictionary.

[15] Heiss WD, Huber M, Fink GR, Herholz K, Pietrzyk U, Wagner R (1992). Progressive derangement of periinfarct viable tissue in ischemic stroke. J Cereb Blood Flow Metab.

[16] Hossmann KA (1994). Glutamate-mediated injury in focal cerebral ischemia: The excitotoxin hypothesis revised. Brain Pathol.

[17] Mak S, Egri Z, Tanna G, Colman R, Newton GE (2002). Vitamin C prevents hyperoxia-mediated vasoconstriction and impairment of endothelium-dependent vasodilation. Am J Physiol Heart Circ Physiol.

[18] Hyperbaric oxygen chamber treatment centers

[19] Central Medico-Sanitary Division

[20] Zubkov YN, Nikiforov BM, Shustin VA (1984). Balloon catheter technique for dilation of constricted cerebral arteries after aneurysmal SAH. Acta Neurochir (Wien).

[21] Kaku Y, Yonekawa T, Kazekawa K (1992). Superselective intra-arterial infusion of papaverine for the treatment of cerebral vasospasm after subarachnoid hemorrhage. J Neurosurg.

[22] Feng L, Fitzsimmons BF, Young WL, Berman MF, Lin E, Aagaard BD (2002). Intraarterially administered verapamil as adjunct therapy for cerebral vasospasm: Safety and 2-year experience. AJNR Am J Neuroradiol.

